# Alternative Splicing of hTERT Pre-mRNA: A Potential Strategy for the Regulation of Telomerase Activity

**DOI:** 10.3390/ijms18030567

**Published:** 2017-03-07

**Authors:** Xuewen Liu, Yuchuan Wang, Guangming Chang, Feng Wang, Fei Wang, Xin Geng

**Affiliations:** 1 Department of Biochemistry and Molecular Biology, School of Basic Medical Sciences, Tianjin Medical University, Tianjin 300070, China; superryan2016@163.com; 2 Key Laboratory of Immune Microenvironment and Disease (Ministry of Education), Tianjin Medical University, Tianjin 300070, China; 3 Tianjin Key Laboratory of Ophthalmology and Visual Science, Tianjin Eye Hospital, Tianjin 300070, China; wyc_81@126.com; 4 Clinical College of Ophthalmology, Tianjin Medical University, Tianjin 300070, China; 5 Department of Clinical Laboratory, General Hospital, Tianjin Medical University, Tianjin 300070, China; szxiaoming123@163.com; 6 Department of Genetics, Tianjin Medical University, Tianjin 300070, China; wangf@tmu.edu.cn; 7 Department of Neurology, General Hospital, Tianjin Medical University, Tianjin 300052, China

**Keywords:** telomerase, human telomerase reverse transcriptase (hTERT) pre-mRNA, alternative splicing, cancer, aging

## Abstract

The activation of telomerase is one of the key events in the malignant transition of cells, and the expression of human telomerase reverse transcriptase (hTERT) is indispensable in the process of activating telomerase. The pre-mRNA alternative splicing of hTERT at the post-transcriptional level is one of the mechanisms for the regulation of telomerase activity. Shifts in splicing patterns occur in the development, tumorigenesis, and response to diverse stimuli in a tissue-specific and cell type–specific manner. Despite the regulation of telomerase activity, the alternative splicing of hTERT pre-mRNA may play a role in other cellular functions. Modulating the mode of hTERT pre-mRNA splicing is providing a new precept of therapy for cancer and aging-related diseases. This review focuses on the patterns of hTERT pre-mRNA alternative splicing and their biological functions, describes the potential association between the alternative splicing of hTERT pre-mRNA and telomerase activity, and discusses the possible significance of the alternative splicing of the hTERT pre-mRNA in the diagnosis, therapy, and prognosis of cancer and aging-related diseases.

## 1. Introduction

Human telomerase reverse transcriptase (hTERT), the catalytic subunit of the telomerase holoenzyme complex, plays an important role in cell senescence and tumorigenesis. The molecular mechanisms of the regulation of telomerase are fairly complicated, including various levels such as transcription, post-transcription, post-translation and sub-cellular localization [1,2]. hTERT promoter mutation, for instance, is one of the pathways of the regulation mechanisms. Investigations on cancer-specific TERT promoter mutations help us to understand the mechanistic basis of the activation of the dormant TERT promoter in cancers [3,4].

Another key point of telomerase regulation is the alternative splicing of hTERT pre-mRNA. Alternative splicing, one of the mechanisms for the diversity of proteins, refers to the process of the precursor message RNA (pre-mRNA) transcripted from eukaryotic genes producing different alternative splicing variants (ASVs) in different splicing ways. Different hTERT ASVs code proteins with different functions, and the alternative splicing at the post-transcription level can regulate the expression of genes [5]. Because the processing of hTERT pre-mRNA yields catalytically inactive ASVs, the correlation between hTERT expression and telomerase activity is complicated [6]. So far, more than 20 different hTERT ASVs have been reported, some of which are widely expressed while others show a tissue- or cell type-specific character [7]. Evidence exists that the alternative splicing of hTERT pre-mRNA may play a critical role in the regulation of telomerase activity [8]. Research on hTERT pre-mRNA alternative splicing has started to increase our understanding of the regulation mechanism of telomerase activity, as well as provide useful information for the diagnosis and therapy of cancer and aging-related diseases.

## 2. Alternative Splicing of hTERT Pre-mRNA

The hTERT gene spans 42kb and contains 16 exons and 15 introns. The full-length of hTERT mRNA is approximately 4.0kb. The hTERT protein contains seven reverse transcriptase motifs and one telomerase-specific T motif, which are mostly located on individual exons. The existence of the motifs is the premise of the hTERT functions [9]. The majority of the junctions of introns and exons show conservative 5′- and 3′-splice consensus sequences [10]. At least seven selective splicing sites are located on the hTERT pre-mRNA, including three deletion sites (α, β, and γ) and four insertion sites [11]. Any splicing occurring in different combinations of these sites will produce a number of hTERT ASVs at different levels. However, only the full-length hTERT mRNA with neither the deletion nor insertion of splicing shows telomerase activity [12].

**Figure 1:**
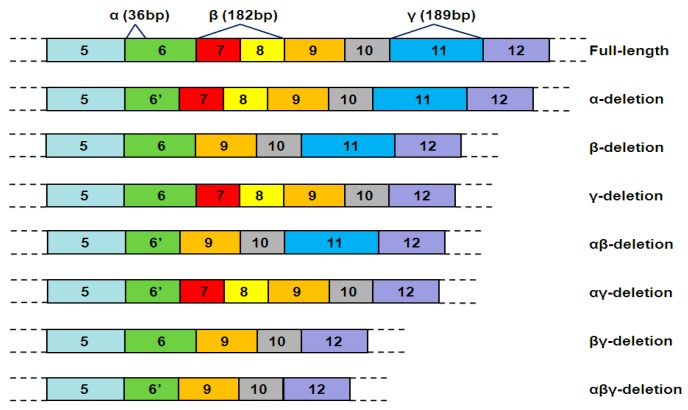
The three deletion sites of human telomerase transcriptase (hTERT) pre-mRNA alternative splicing (α, β, γ). There are seven alternative splicing variants (ASVs) of hTERT, and they are the α-deletion, β-deletion, γ-deletion, αβ-deletion, αγ-deletion, βγ-deletion, and αβγ-deletion ASV. The numbers from 5 to 12 indicate exon 5 to exon 12.

Some of these hTERT ASVs and their functions have been identified. The deletion of 36 nucleotides in exon6, the α splicing site, removes most of the reverse transcriptase motif A. The β-deletion ASV results from the deletion of 182 nucleotides of exon7 and exon8. The deletion of 189 nucleotides inexon11, the γ splicing site, removes sequences that encode motifs D and E [13,14] (Figure 1). The α-deletion ASV and γ-deletion ASV are in-frame deletions, whereas the others are out-of-frame deletions [14]. Moreover, splicing occurring at the β-deletion site will lead to the emergence of a termination codon, causing an early termination of the translation [9]. The γ-deletion ASV and the combination ASVs of the αγ, βγ, and αβγ deletion were of very low intensities [14] (Table 1).

## 3. Alternative Splicing of hTERT Pre-mRNA in the Development of Human Embryo and Human Embryonic Stem Cells

Studies on the alternative splicing of hTERT pre-mRNA in human embryonic tissues have a long history. Ulaner et al. [15] found that only the full-length hTERT mRNA could express telomerase activity in the process of the development of fetus kidney tissue. The telomerase activity would be suppressed at the 15th gestational week. At the same time, hTERT ASVs started to express and were sustained to the 21st gestational week, indicating that the alternative splicing of hTERT pre-mRNA could suppress the activity of telomerase in the development of embryonic kidney tissue. They continued to study the expression of hTERT ASVs in the development of the embryo. They found the alternative splicing patterns of hTERT pre-mRNA were different in different tissues and at different gestational ages [11,15]. The hTERT ASV with 159 bp insert sequences and the αβ-deletion hTERT ASV were only expressed in the embryonic liver. The α-deletion hTERT ASV was expressed significantly in liver tissue and early kidney tissue, while the β-deletion hTERT ASV was expressed remarkably in the liver, kidney, and cardiac tissue of the early embryo. The late embryonic kidney tissue expressed the β-deletion hTERT ASV only, but it was not related to the telomerase activity or the maintenance of the telomere length. The specific expression of hTERT ASVs in the embryonic development indicated that the alternative splicing of hTERT pre-mRNA was not random and each pattern of hTERT ASVs has specific biological functions [11,15].

Betts et al. [16] conducted an in vitro experiment to confirm the role of micro-environmental oxygen tension in mediating the alternative splicing pattern of hTERT pre-mRNA in the development and characteristics of human embryonic stem cells (hESCs). A reverse transcription polymerase chain reaction (RT-PCR) analysis showed the transcript levels of full-length hTERT mRNA and ASVs of TERT in hESCs cultures with varying O_2_ tensions. Constitutively spliced full-length TERT, as well as the α-deletion, β-deletion, and αβ-deletion hTERT ASVs were expressed in H9 hESCs cultures in both high-O_2_ (20%) and low-O_2_ (2%) conditions. However, both the full-length TERT and the α-deletion ASV abundance were significantly reduced in the low-O_2_ condition compared with the high-O_2_ condition. They utilized steric-blocking morpholino antisense oligonucleotides (MO) to reveal the potential roles for the specific TERT ASVs in hESCs. In addition, the results verified that the MO inhibition of specific TERT ASVs could promote spontaneous hESCs differentiation and appeared to be biased toward particular lineages.

## 4. Expression, Distribution and Biological Functions of hTERT ASVs in Cancer and Other Diseases

The role of hTERT ASVs in the process of the regulation of telomerase activity still remains unclear. Researchers have been striving to explore the molecular mechanisms by using various qualitative and quantitative measurements of hTERT ASVs.

Woodring E. Wright and his team chose several strains of normal cells, immortalized cells, and tumor cells to detect the expression of hTERT ASVs by use of RT-PCR [13]. While hTERT ASVs have been found in a large number of cell types, the spectra and relative proportions of hTERT ASVs differ [15,17]. The β-deletion ASV was the most abundant and widely expressed isoform of the full-length hTERT mRNA, whereas the α-deletion ASV tended to be weakly expressed [17].

It was verified that the hTERT γ-deletion ASV was expressed in gastric cancer cell lines and hepatocellular carcinoma cell lines, but not in colorectal cancer cell lines. It indicated that the expression of hTERT ASVs might be tissue-specific and/or cell type-specific [18].

However, Barclay et al. [19,20] detected telomerase activity and the expression of TERT transcripts in esophageal and gastric adenocarcinoma, with the adjacent normal mucosa tissue as a control. Their results showed a significant correlation between hTERT mRNA levels and telomerase activity in gastric adenocarcinoma and normal mucosa samples, without significant differences in the patterns and expression levels of all hTERT ASVs observed in the cancer tissues, indicating that hTERT ASVs did not appear to play a role in the regulation of telomerase activity during tumorigenesis in these tissues.

A novel real-time PCR protocol was developed for the quantification of telomerase and three main hTERT ASVs to test the correlation between them in lung cancer [21]. The results showed that lung carcinoma cell lines consistently expressed various hTERT ASVs and telomerase activity. It was not exact to describe the association of the total TERT with telomerase activity as a significant correlation. The telomerase activity correlated with the transcriptional constituent ratio of α-deletion, β-deletion and γ-deletion ASVs, among which the positive rate and constituent ratio of β-deletion ASVs were higher than the other two isoforms. The relative lower level of expression of β-deletion ASVs in combined small-cell and squamous cell carcinomas might explain their higher telomerase activity.

Researchers from Johns Hopkins Medical Institutions implemented a study to investigate the relationship between telomere length and hTERT ASV expression patterns in benign and differentiated malignant thyroid tumors. A greater proportion of full-length hTERT over the ASVs was found in malignant tumors compared with their benign counterparts, while no association between the telomerase activity and levels of the individual ASV was found, other than the wild type. The α-deletion hTERT ASV resulted in a dominantly negative regulation of telomerase activity. The β-deletion and αβ-deletion hTERT ASVs produced shortened nonfunctional proteins because of the premature stop codons [22].

Telomerase activity and hTERT ASV expression has been reported in 27 endometrium, 14 myometrium, and 18 endometrial cancers. Telomerase activity and full-length hTERT mRNA was expressed in the endometrium proliferation and early secretion stage, but not in the late secretion stage. Endometrium was expressed as a constant β-deletion ASV in the whole menstrual cycle. The β-deletion ASV was widely expressed in the myometrium, while only three samples expressed telomerase activity in the study [23].

The differences were compared in the expression of hTERT ASVs and telomerase activity in unstimulated T-cells among myelodysplastic syndrome (MDS) subgroups and healthy controls. The results showed that the telomerase activity, total hTERT mRNA level, and β-deletion ASV expression level in unstimulated T-cells from MDS samples were significantly higher than those from the controls. Other hTERT ASVs were lower in expression and there were no significant differences among MDS subgroups and controls. The telomerase activity was correlated with the total hTERT levels positively in MDS (*r* = 0.58, *p* = 0.007) [24].

Researchers from the Cancer Center Karolinska [25] demonstrated in their report that hTERT ASVs in chronic lymphocytic leukemia were associated with disease activity, the clinical stage, and the mutational status of immunoglobulin heavy chain variable (IGHV) genes’ mutational status. A higher level of the α-deletion ASV was observed in un-mutated IGHV gene patients compared with those in mutated patients, whereas no difference was detected for the β-deletion ASV. Patients, whether they had mutated IGHV genes or not, were found to have lower expression levels of the β-deletion ASV compared with the controls.

Terrin et al. [26] investigated hTERT gene expression in 134 B-cell chronic lymphocytic leukemia (B-CLL) cases and evaluated its prognostic value with other prognostic markers, such as immunoglobulin V (IgVH) mutation status, and CD38 and ZAP-70 expression. They discovered that levels of all the hTERT ASVs were strongly correlated with the full-length hTERT, both of which inversely correlated with the percentage of IgVH mutation and were significantly higher in unmutated than in mutated cases.

The function of the hTERT ASVs in regulating telomerase activity has been studied in detail in cancer cell lines. Yi et al. [13] reverse-transcribed the α-deletion ASV, β-deletion ASV, and αβ-deletion ASV respectively into their corresponding cDNAs and then linked the cDNAs with reverse transcription virus vectors to transfect the normal human fibroblast and several strains of telomerase-positive immortalized cells and tumor cells. The results showed that all the three patterns of hTERT ASVs could not rebuild the telomerase activity of the human fibroblast. The over expression of the α-deletion ASV in the immortalized cells or tumor cell strains (SW39, H1299, DU145), although unable to abolish telomerase activity completely, led to the suppression of telomerase activity and shortened the telomere length gradually and ultimately resulted in the apoptosis of SW39 and DU145. However, no detectable effect on the telomerase activity of the β-deletion ASV and αβ-deletion ASV was found. Thus they concluded that the α-deletion ASV had a dominant negative effect on telomerase activity. The study of Colgin et al. [27] also verified that the α-deletion ASV could suppress endogenous telomerase activity, which resulted in the shortening of telomere and chromosome end-to-end fusion. The suppression of telomerase led to the senescence of HT1080 cells and the apoptosis of the jejunum fibroblast. The down-regulative effect of the α-deletion ASV on the telomerase activity might be dose-dependent, while the lack of in vitro experiments with a stable antibody prevents verification of this. It might be viable to up-regulate the α-deletion ASV to suppress telomerase activity by way of controlling the alternative splicing of hTERT pre-mRNA.

**Figure 2:**
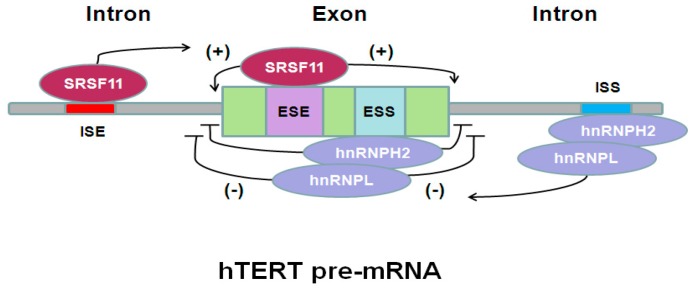
Control of hTERT pre-mRNA alternative splicing. SRSF11 (one of the serine-arginine repeat SR proteins) can act to promote splicing by interacting with the splicing enhancers. hnRNPH2 and hnRNPL (two proteins belonging to the heterogeneous nuclear ribonucleoprotein particle hnRNP family) can act to inhibit splicing by acting with the splicing silencers. ESE: exonic splicing enhancer; ESI: exonic splicing silencer; ISE: intronic splicing enhancer; ISS: intronic splicing silencer.

Unlike the α-deletion ASV, the β-deletion ASV was widely and highly expressed in stem and cancer cell lines [9,12]. Blackburn et al. [12] demonstrated in their research report that the β-deletion ASV coded for a truncated protein, reserving the known RNA-binding motifs other than most of the reverse transcriptase domains. In the breast cancer cells, the splicing pattern of the β-deletion ASV was controlled by the splicing regulators SRSF11, hnRNPH2, and hnRNPL. When over-expressed ectopically, the β-deletion protein localized to the nucleus and mitochondria, competed to bind to the human telomerase RNA (hTR), and thereby inhibited the endogenous telomerase activity as protecting breast cancer cells from apoptosis (Figure 2).

## 5. hTERT Pre-mRNA Alternative Splicing in the Diagnosis and Prognosis of Cancer

About 85% of cancer cells show telomerase activity, which is rarely detectable in somatic cells [28]. High telomerase activity has been shown to be a prognostic marker connected with a poor clinical outcome in several cancer types [29,30,31]. hTERT ASVs have been proposed to represent both diagnostic and prognostic biomarkers in cancer patients, and have been correlated with tumor histopathological and clinical parameters [12,32,33]. The telomerase activity is commonly measured by the use of a telomeric repeat amplification protocol (TRAP) assay [34].

In a study on several lung cancer cell strains, Fujiwara et al. [35] found that both TKB-4 cells and TK-20 cells expressed hTERT mRNA but in different splicing patterns: the former was telomerase-positive and the latter was telomerase-negative. In TKB-4 cells, the proportion of full-length hTERT mRNA was 15.4% of the total hTERT mRNA. However, the TK-20 cells showed a remarkable expression of the β-deletion ASV but almost no full-length hTERT mRNA, indicating that the hTERT pre-mRNA alternative splicing did have effects on the telomerase activity. Therefore, the hTERT pre-mRNA alternative splicing should be taken into account when measuring the hTERT mRNA expression.

In the study of Blackburn et al. [12], higher telomerase activity in basal subtypes of breast cancer was accompanied by a higher percentage of expression of full-length hTERT mRNA. Conversely, lower telomerase activity in luminal subtypes correlates with higher levels of the β-deletion ASV. Evidence could also be found in a study by Mellstedt et al. in patients of chronic myelogenous leukemia; in this study, levels of both full-length hTERT mRNA and β-deletion ASVs were higher in progressive than in non-progressive patients [25].

It has been reported that tumors with a low level or no telomerase activity only express shortened hTERT ASVs, while tumors with a high telomerase activity always express full-length hTERT mRNA [36]. Researchers conducted a retrospective study on 124 neuroblastoma patients, and detected the expression of hTERT ASVs in formalin-fixed and paraffin-embedded samples by RT-PCR. They also compared the results with clinical follow-up information. It turned out to be feasible to detect hTERT mRNA expression in histopathological slices by RT-PCR. Compared with the prognosis factor of neuroblastoma, the full-length hTERT mRNA was an independent and effective prognosis factor [36].

## 6. hTERT Pre-mRNA Alternative Splicing Regulation in Cancer Therapy

Significant efforts have been made to develop telomerase as a therapeutic treatment for malignant tumors. One feasible strategy to develop effective therapies would be to regulate the alternative splicing of hTERT pre-mRNA patterns to change the constituent ratio of the hTERT transcripts. The telomerase activity will decrease as the active hTERT mRNA expression level declines. It is a possible viable approach to manipulate some splicing factors or degradation of hTERT ASVs in order to reduce the telomerase activity to render inhibition of the enzyme [37,38].

It has been reported that ligand 12459 (triazine derivative) was capable of regulating the splicing pattern of hTERT pre-mRNA. The administration of 12459 on A549 lung cancer cells had no obvious effect on the expression of the total hTERT mRNA, while functional hTERT mRNA was almost undetectable and the β-deletion ASV increased. The effect appeared 24 h after administration, and peaked at 48 h. The effect was sustained for up to 72 h without increased efficacy. The hTERT intron 6contains two sequences rich in G to form G-quadruplex structures which might combine with some splicing factors regulating hTERT exon 7 and exon 8 negatively. It was identified that 12459 could stabilize the G-quadruplex structure, resulting in the dysfunction of the splicing factors. The splicing of exon 7 and exon 8 regenerated and led to the reduction of the functional hTERT mRNA level. Then the expression level of the β-deletion ASV increased. The G-quadruplex structures also localized at the single strain of the telemetric terminal overhang. Another way for 12459 to inhibit telomerase activity might be to stabilize the formation of G-quadruplex structures on the telomere ends [39].

The chemically modified antisense oligonucleotides were reported to also potentially change the splicing patterns of hTERT pre-mRNA. The combination of 2′-*O*-methyl-Phosphorothioates (2′-*O*-methyl-PTOs) with the target RNAs formed double strains to block the degradation of RNase H and other RNases. The designed 2′-*O*-methyl-PTOs complementarily combined on the splicing site located at the junction of intron 5 and exon 6 in the hTERT pre-mRNA. The RT-PCR was used to detect the expression levels of all the hTERT transcripts after the administration of the oligoribonucleotide to the DU145 human prostatic cancer cells. The results showed that the functional transcript decreased while the α-deletion ASV and αβ-deletion ASV increased. The β-deletion ASV maintained its high expression level. The change in the hTERT pre-mRNA splicing pattern consequently led to a remarkable suppression of telomerase activity, cell growth, and apoptosis [40].

Another telomerase inhibitor, imetelstat (GRN163L), showed activity in various cancers and has entered Phase I and II clinical trials. However, it still remains unclear if imetelstat as a single factor can achieve sustained inhibition in patients. Immunotherapy was also a potential therapeutic option for telomerase that is primarily active in cancer cells. To generate a telomerase-targeted immunotherapy, TERT was presented as the human leukocyte antigen (HLA) class I to cytotoxic T cells (CD8+) to motivate them, leading to the lysis of cancer cells. By this method, up to 26 different TERT peptides have been made to elicit an effective anti-tumor response. However, stem cells and germ cells could also be affected because both of them express TERT as well [41].

The preceding sections demonstrated the role of telomerase alternative splicing in embryonic development and tumors. The current accurate estimate for the quantity of catalytically active telomerase molecules is 50–100 per cell [42], produced from approximately 20 mRNA molecules per cell. The minority of the hTERT transcripts were spliced into the full-length isoform with telomerase activity [43]. A working explanation of these results was that the transcriptional machinery was not able to reduce the transcription to a level that produces only a few mRNA molecules within a single cell. The excess transcripts then underwent alternative splicing into nonfunctional isoforms to maintain the low level of telomerase. The splicing of hTERT was regulated by long-range interactions instead of the intronic/exonic elements adjacent to the splicing sites. Almost nothing was known about the regulation mechanisms of low abundant proteins such as telomerase, even though it has been identified for nearly 20 years [38,43].

## 7. Conclusions and Prospects

Plenty of studies in the past decades have revealed that telomerase activity was involved in critical mechanisms in the genesis and progression of tumors. The main role of telomerase in tumorigenesis is to maintain the length of the telomere. Although only the full-length hTERT mRNA is strongly associated with telomerase activity, the alternative splicing of hTERT is also verified to be correlated with histopathological and clinical parameters of tumors. However, the exact regulation mechanism and biological functions of hTERT are still uncertain. As technology advances, the understanding of hTERT alternative splicing has increased tremendously. Future studies are expected to identify novel splicing sites, regulation, and the mechanisms of splicing. It is doubtless that the alternative splicing of hTERT pre-mRNA will have a role in anti-cancer therapies in the future.

## Figures and Tables

**Table 1:** Information of hTERT mRNA alternative splicing variants.

Typical hTERT mRNA Variants	Telomerase Activity	Tissue-Specificity	Functions
Full-length hTERT mRNA	Yes	No	promote telomerase activity
α-deletion ASV	No	Yes	Inhibit telomerase activity
β-deletion ASV	No	Yes	Inhibit telomerase activity but protect from apoptosis
γ-deletion ASV	No	Yes	Inhibit telomerase activity
αβ-deletion ASV	No	Yes	Unclear
αγ-deletion, βγ-deletion and αβγ-deletion ASV	No	Unclear	Unclear
Δ4–13	No	Unclear	Extra-telomeric functions, stimulate cell proliferation
Insertion ASVs	No	Unclear	Unclear
